# Bidirectional Optogenetically-Induced Plasticity of Evoked Responses in the Rat Medial Prefrontal Cortex Can Impair or Enhance Cognitive Set-Shifting

**DOI:** 10.1523/ENEURO.0363-19.2019

**Published:** 2020-01-02

**Authors:** Sarah E. Bulin, Kelly M. Hohl, Denisse Paredes, Jeri D. Silva, David A. Morilak

**Affiliations:** 1 Department of Pharmacology and Center for Biomedical Neuroscience, University of Texas Health San Antonio, San Antonio, TX 78229; 2 South Texas Veterans Health Care System, San Antonio, TX 78229

**Keywords:** chronic stress, cognitive flexibility, medial prefrontal cortex, neuroplasticity, optogenetics, set-shifting

## Abstract

Chronic stress compromises cognition, including executive function mediated in the medial prefrontal cortex (mPFC). To investigate mechanisms underlying these processes, we use chronic unpredictable stress (CUS), which reduces activity in the mPFC and impairs cognitive set-shifting, a measure of cognitive flexibility in laboratory rats. It has been shown that CUS attenuates the local electrical field potential response evoked in the mPFC by stimulation of the ascending excitatory afferent from the mediodorsal thalamus (MDT). Thus, in this study, to investigate the role that such changes in afferent-evoked responsivity of the mPFC might play in the cognitive deficits induced by CUS, we used optogenetics to directly induce plastic changes in the thalamic-mPFC afferent pathway. Glutamatergic neurons in the MDT were virally-induced to express the ChETA variant of channelrhodopsin. Then, to first validate the optogenetic induction of plasticity, long-term depression (LTD) or long-term potentiation (LTP) were induced by laser stimulation of ChETA-expressing terminals in the mPFC of anesthetized rats. In subsequent experiments, induction of opto-LTD in awake animals produced set-shifting deficits similar to those induced by CUS. By contrast, inducing opto-LTP in rats that had received prior CUS treatment corrected the stress-induced deficit in set-shifting. These results suggest that stress-induced plasticity in the thalamic-mPFC pathway is sufficient to produce stress-induced cognitive deficits, and may represent a novel target for effective therapeutic intervention to correct cognitive impairment in stress-related psychiatric disorders.

## Significance Statement

Chronic stress reduces the response of the medial prefrontal cortex (mPFC) to afferent input, and also impairs cognitive flexibility mediated by the mPFC. We investigated if plasticity in the response of the mPFC to input from the thalamic afferent originating in the region of the mediodorsal thalamus (MDT) could account for stress-induced changes in cognitive capability. Direct optogenetic depression of the thalamic-mPFC pathway impaired cognitive set-shifting similarly to chronic stress. By contrast, directly potentiating this pathway optogenetically restored set-shifting performance in stressed rats. Thus, plasticity in the thalamic-mPFC pathway may be a mechanism for stress-induced cognitive impairment, and restoring function in this pathway may be an effective strategy for therapeutic intervention.

## Introduction

Cognitive flexibility, an executive process mediated by the medial prefrontal cortex (mPFC), is compromised by chronic stress. Deficits in cognitive flexibility impair an individual’s ability to modify established thought patterns or behavioral strategies based on changing environmental feedback (Merriam et al., 1999; Birrell and Brown, 2000; Stuss et al., 2000). Stress is a risk factor for anxiety and mood disorders, and impaired cognitive flexibility is a characteristic of these disorders. Along with other deficits in executive function, impaired cognitive flexibility may not only represent a symptom of these illnesses, but may contribute causally to their development and maintenance (Vasterling et al., 1998; Coles and Heimberg, 2002; Rogers et al., 2004). The mPFC is hypoactive in patients suffering from disorders involving impaired cognitive flexibility, such as posttraumatic stress disorder (PTSD) or depression (Matsuo et al., 2003; Sheline, 2003; Rogers et al., 2004). Notably, mPFC activity is normalized in patients that receive effective behavioral therapy (Yoshimura et al., 2014). Congruently, preclinical studies have demonstrated that chronic unpredictable stress (CUS) impaired cognitive flexibility and attenuated electrical responses evoked in the mPFC by stimulating the excitatory afferent from the mediodorsal thalamus (MDT; Bondi et al., 2008; Fucich et al., 2018). Further, a rat model of behavioral therapy by extinction training effectively restored the afferent-evoked mPFC response and reversed the cognitive set-shifting deficit in stressed rats (Fucich et al., 2016, 2018).

Evidence suggests that stress-induced hypoactivity of the mPFC may represent a pathway-specific dysregulation, rather than an overall decrease in mPFC function. After CUS, the mPFC was less responsive to stimulation from the MDT, but the response to ventral hippocampal afferent activation remained unchanged, indicating a pathway-specific deficit (Jett et al., 2017). Glutamatergic neurons of the MDT project to pyramidal neurons in the mPFC, with the densest innervation of neurons in layer II/III (Groenewegen, 1988; Kuroda et al., 1996). Chronic restraint stress reduced dendritic arborization of pyramidal neurons within these same layers of the mPFC (Liston et al., 2006), which may result in reduced excitatory synaptic connections in the thalamic-mPFC pathway after stress. The MDT is also involved in cognitive flexibility, as direct inactivation of the MDT increased the number of trials required to reach criterion in a set-shifting test (Block et al., 2007). Thus, stress-induced plasticity in the thalamic-mPFC pathway may be a mechanism by which chronic stress can produce cognitive deficits, and may therefore represent a potential target for therapeutic intervention in stress-related psychiatric disorders.

In the present study, we hypothesized that CUS-induced deficits in cognitive set-shifting are attributable to attenuated responsiveness of the mPFC to afferent input from the thalamus. Using the extradimensional (ED) set-shifting task of the attentional set-shifting test (AST) as a measure of cognitive flexibility mediated by the mPFC, we tested this hypothesis by directly and specifically depressing or potentiating the glutamatergic thalamic-mPFC afferent pathway optogenetically. Glutamatergic neurons in the MDT were induced by viral infection to express the ChETA variant of channelrhodopsin, driven by a CamKIIα promoter. We first confirmed that laser stimulation of thalamic afferent terminals in the mPFC, using stimulus parameters resembling those used to produce long-term depression (LTD) or long-term potentiation (LTP) electrically, produced changes in afferent-evoked response resembling electrically-induced LTD or LTP specifically in the thalamic-mPFC pathway. We also tested whether CUS occluded the induction of LTD, suggesting that similar mechanisms may be involved in both. We next induced opto-LTD in the thalamic-mPFC pathway before performance on the set-shifting task to test the hypothesis that reducing synaptic strength in this pathway in unstressed animals will mimic the cognitive deficits seen after CUS. We then tested the hypothesis that directly potentiating responses in the thalamic-mPFC pathway by inducing opto-LTP would be sufficient to restore set-shifting in animals exposed to CUS.

## Materials and Methods

### Animals

A total of 61 male (200–220 g on arrival) and 78 female (180–200 g on arrival) Sprague Dawley rats (Envigo, RRID:RGD_737903), were used for these studies. Before beginning any experimental procedures, rats were individually housed in 25 × 45 × 15 cm cages and maintained on a 12/12 h light/dark cycle (lights on at 7 A.M.). Individual housing was maintained throughout the experiment to prevent animals from chewing on each other’s healing postsurgical wounds, to ensure consistent food intake during periods of food restriction for AST, and because social housing mitigates the effects of stress. All procedures were conducted during the light phase, with food and water ad libitum except during the period of food restriction before testing on the AST. For the social defeat stressor in the male CUS protocol, 12 Long–Evans retired male breeders were each pair-housed with an ovariectomized female (Charles River, RRID:RGD_2308852) in large cages (63 × 63 × 40 cm) in a separate room. All procedures were approved by the University of Texas Health, San Antonio, Animal Care and Use Committee and were consistent with National Institutes of Health guidelines.

### Viral delivery of ChETA or GFP-control constructs

Glutamatergic neurons in the MDT were infected with an AAV5 virus carrying a construct encoding the ChETA variant of blue light-sensitive channelrhodopsin (Gunaydin et al., 2010) driven by a CamKIIα promoter for selective expression in glutamatergic neurons, or a GFP control construct. Rats were anesthetized with isoflurane and placed in a stereotaxic apparatus. A microinjector was lowered into the MDT (coordinates from bregma: AP –2.5 mm; ML ±0.9 mm; DV –4.6 mm), and the AAV5-CaMKIIα-ChETA-YFP or control AAV5-CaMKIIα-GFP virus (3 × 10^9^ viral particles/μl, UPenn Vector Core obtained through Addgene, RRID:Addgene_100050) was injected bilaterally (0.5 μl per side at 0.2 μl/min). Injectors were left in place for 10 min to allow for diffusion before withdrawing. Injections were centered in the MDT, but because of the small size of the MDT target, and because it was impossible to specify the extent of viral uptake six to seven weeks after injection, it cannot be ruled out that other nearby thalamic areas may also have been included in the spread of injection and viral uptake. Thus, we will refer to the targeted pathway more generally as the “thalamic-mPFC afferent.”

For rats in experiments 2 and 4, after virus was injected, dual fiber optic cannulae (Doric Lenses) were implanted 1.0 mm above the infralimbic (IL) cortex in the ventral mPFC (AP +3.0 mm; ML ±0.6 mm; DV –3.5 mm) and secured to the skull using jeweler’s screws and dental cement. Animals began behavioral training six weeks after surgery to ensure expression and transport of the channel to terminals innervating the mPFC.

### CUS

A different acute stressor was administered once daily (Table 1). Based on the results of experiment 1, CUS was administered for two weeks for males and three weeks for females, to achieve the same set-shifting deficit in both. Descriptions of individual stressors are as follows. Electric footshock consisted of 1.5-mA scrambled shock delivered through the grid floor of a shock chamber. The shock was applied 5 s on, 175 s off, for 15 min. For 30-min restraint, rats were placed in a restraining device made of Plexiglas and flexible nylon, which was closed using two Velcro strips, thus restricting movement but allowing free respiration and air circulation. For social defeat, the “intruder” Sprague Dawley test rat was transported to the Long–Evans residents’ housing room. The ovariectomized female was removed from the resident cage immediately before introducing the intruder. Within 2–5 min, the intruder was attacked and defeated, indicated by fleeing, freezing, and submissive behavior. When full submissive posture occurred, it was separated from the resident, protected from further attacks by placing it under a small wire mesh cage (30 × 15 × 15 cm), and kept in the resident cage for 45 min. This allowed for continuing auditory, olfactory, and visual contact between the two rats. The defeated rat was then removed and returned to its home cage and the Long–Evans female returned to the resident cage. The shaking/overcrowding procedure was conducted by placing six rats in a plastic bin on a lab shaker set to produce 220 back-and-forth movements per min. Tail pinch involved placing the rat in the previously described restraining device, and applying a clothespin 1 cm from the base of the tail for 10 min. Lights on consisted of placing the rat’s home cage in a room with the lights on overnight, and returned to normal housing the next day. For, wet bedding the rat was placed in a cage with ∼0.5 inches of water in the bottom of the cage to saturate the bedding overnight. The next morning the rat was returned to a fresh cage. Additional modification of the CUS procedure for females is described below in the methods for experiment 1. Following each stressor, rats were placed in an isolated room to recover for 1 h, then transferred to a clean cage and returned to housing. Unstressed controls remained in housing and were handled 1–2 min/d for the same period of time before behavioral testing.

**Table 1.** CUS schedule for male and female rats

CUS procedure		
Day	Males	Females
1	15-min footshock	15-min footshock
2	30-min restraint	30-min restraint
3	Social defeat	24-h lights on
4	10-min tail pinch	10-min tail pinch
5	24-h wet bedding	24-h wet bedding
6	15-min footshock	15-min footshock
7	1-h shake/overcrowding	1-h shake/overcrowding
8	Social defeat	24-h wet bedding
9	10-min tail pinch	10-min tail pinch
10	24-h lights on	24-h lights on
11	Social defeat	15-min footshock
12	10-min tail pinch	10-min tail pinch
13	24-h lights on	24-h lights on
14	15-min footshock	30-min restraint
15		24-h wet bedding
16		15-min footshock
17		10-min tail pinch
18		24-h lights on
19		1-h shake/overcrowding
20		24-h wet bedding
21		15-min footshock

Rats were exposed to one daily stressor over a series of 14 d (males) or 21 d (females). Individual stressor protocols described in further detail in the methods section.

### AST

Rats tested on the AST were food restricted (14 g/d for males; 7 g/d for females) for one week before testing. In the testing arena (75 × 44 × 30 cm), a removable divider formed a start gate in the proximal third of the arena. A Plexiglas divider separated the distal third of the arena into two sides, each containing a terracotta digging pot (diameter 7 cm, depth 6 cm). The pots were differentiated by cues within two distinct stimulus dimensions: the texture of the digging medium that filled the pot, and an odor with which each pot was scented by applying aromatic oil to the rim (Frontier Natural Brands). On day 1 after the end of CUS (habituation), rats were taught to dig for a food reward, a ¼ piece of Honey Nut Cheerio (General Mills Cereals), buried in pots filled with sawdust. On day 2 (training), in the testing arena, rats learned to retrieve the food reward buried in the bottom half of one pot. The rat was placed in the start box, and the gate was lifted to start a trial. The rat was allowed to explore both pots before choosing to dig in one. To prevent location of the reward by smell, the digging media in both pots were lightly dusted with Cheerio powder. Rats were trained to make two simple discriminations (SDs) in the arena. Reward was first associated with an odor (i.e., lemon vs rosewood, pots filled with sawdust), then with a digging medium (i.e., shredded felt vs shredded paper, pots unscented). All rats were trained using these same stimuli. The stimuli used during training were not used again during testing. On day 3 (testing), rats were tested on a series of discrimination tasks, in which the discriminative stimulus dimension and positive cue within that dimension were varied according to a pre-set contingency schedule. When the test subject mastered a given task, indicated by reaching a criterion of six consecutive correct trials, the contingency was changed and they proceeded to the next task in the sequence. The first task was a SD, similar to the training tasks. Half the rats discriminated between pots differentiated by odor, and half between digging media in unscented pots (for clarity, the following description will consider the example beginning with odor as the discriminating stimulus). The second task was a compound discrimination (CD), in which odor remained the informative dimension, and the second, irrelevant dimension (e.g., medium) was introduced as a distractor. The third task was a reversal (R1), in which the same odors and media were used, but the previously positive cue was now negative and the previously negative cue was positive. The fourth task was an intradimensional (ID) shift, i.e., a new acquisition in which all new media and odors were introduced, and odor remained informative. The fifth task was a second reversal (R2). The sixth task was the ED set-shift; all new stimuli were again introduced, but this time the relevant dimension was switched to the digging medium, and odor became the distractor. The dependent measure was the number of trials required to reach criterion of six consecutive correct responses on the ED set-shifting task. If no choice was made within a maximum trial time limit of 10 min, the trial was omitted and scored as a “no choice” trial. If six no choice trials occurred in a row (i.e., 1 h without a dig), the rat would be removed from the study. Eight rats total were removed after six no choice trails. One rat was removed from the CUS group and the other seven were nonstressed. All rats were removed before any type of laser or no laser treatment was given.

#### Experiment 1: validation of CUS-induced set-shifting deficits in female rats

As CUS-induced deficits in set-shifting have only been reported previously in male rats (Bondi et al., 2008), it was necessary to validate an effective CUS protocol in females. A total of 28 female rats weighing 225–249 g were used for experiment 1. To test whether female rats exhibited deficits similar to males on the set-shifting task after CUS, the first cohort of female rats (*n* = 8–9/group) were exposed to an adapted CUS protocol or control treatment (daily handling) for 14 d. Because of practical difficulties implementing social defeat stress in female rats (e.g., use of lactating females as residents), the social defeat stressors in the standard male protocol were replaced with 15-min footshock and 10-min tail pinch stressors on days 3 and 9, respectively. Testing on the AST took place 3 d after the end of CUS or control treatment, as described above.

Results from the first cohort indicated that females were more resistant to CUS than males, as they did not exhibit set-shifting deficits after the 14-d CUS treatment (see Results). Thus, in a second cohort of females (*n* = 5–6/group), we extended the CUS treatment to 21 d. Further, as it has been reported that chronic stress procedures that include circadian disruption more effectively impact female physiology and measures of anhedonia in females (Konkle et al., 2003), we also added a 24-h continuous light stressor to the CUS protocol (Table 1).

#### Experiment 2: validation of optogenetically-induced plasticity

To confirm that both LTD and LTP could be induced optogenetically, we used *in vivo* electrophysiology to measure changes in local field potentials evoked in the mPFC by MDT stimulation before and after laser stimulation with either the LTD or LTP protocols described below. A total of 16 rats (females, *n* = 6; males, *n* = 10), injected into the MDT with virus containing either the ChETA or control constructs six weeks before recording, were anesthetized using chloral hydrate (400 mg/kg, i.p.) and placed in a stereotaxic apparatus. A bipolar stainless-steel stimulating electrode was lowered into the right MDT (from bregma; AP: −2.5 mm, ML: +0.9 mm, DV –4.6 mm). A tungsten parylene-coated recording electrode (A-M Systems, Sequim WA) was placed in the ipsilateral mPFC (from bregma; AP: −3.0 mm, ML: +0.6 mm, DV –3.5 ± 0.5 mm). An optical fiber affixed to the recording electrode 1 mm above the tip was connected to a 473-nm solid-state laser diode (OptoEngine) with 13- to 15-mW output. Following a 15- to 30-min acclimation period, evoked responses were recorded (low cutoff filter 0.3 Hz, high cutoff 1000 Hz) and digitized (Power Lab; ADInstruments, RRID:SCR_001620). Before establishing baseline, a stimulus-response curve was generated by stimulating the MDT (260-μs pulse width, 0.1 Hz, 100–800 μA) and recording in the mPFC to determine maximum response amplitude. Response amplitude was measured from the peak of the first negative deflection (N1), occurring at a latency of 5 ± 1 ms after stimulation, to the peak of the first positive deflection (P1), at a latency of 18 ± 3 ms. This corresponds with the timing of single cell firing evoked in the mPFC by stimulating this pathway (Herry and Garcia, 2002). Baseline responses were then collected by stimulating once every 10 s at 75% of maximum response for 15 min. Electrical stimulation from the MDT was then turned off and rats underwent optical stimulation protocols for either LTD induction (900 light pulses, 2-ms pulse width at 1 Hz, for a total of 15 min), or LTP induction (10 × 1 s trains of light pulses, 1-ms pulse width at 250 Hz, with 10 s between each train for a total of 1.5 min) within the mPFC. Immediately after induction, electrical stimulation was resumed and responses were recorded for 3 h. Traces were averaged per minute (six traces), and mean baseline was calculated as the average across the entire 15-min baseline period. Response amplitude during each minute following induction was then calculated as a percentage of the mean baseline, and analyzed in 5-min bins.

To test whether opto-LTD and the depression of evoked response after CUS may involve similar mechanisms, we investigated if the induction of opto-LTD was occluded in CUS-treated rats (*n* = 5). As the duration of CUS treatment differed for males and females (Table 1), CUS began three weeks (females; *n* = 2) or four weeks (males; *n* = 3) after viral injection. Three days after the end of CUS, animals were anesthetized for field potential recordings and opto-LTD induction. To confirm that opto-LTP could still be induced after CUS treatment, a separate cohort of CUS-treated rats (females, *n* = 3; males, *n* = 2) was anesthetized for *in vivo* field potential recordings and opto-LTP induction. After completing the experiments, rats were sacrificed and electrode placements confirmed histologically. Any case in which recording electrodes were not located in the mPFC, or stimulating electrodes were not located in the MDT would be eliminated. One CUS-LTP rat was removed for misplacement of the stimulating electrode outside the MDT.

### Immunohistochemistry (IHC) for confirmation of virally-delivered ChETA expression

IHC was performed to confirm ChETA expression both in cells within the injection site in the MDT and in terminals within the mPFC. After AST, rats were sacrificed via rapid decapitation and brains were postfixed in 4% paraformaldehyde. Brains were sectioned at 40 μm on a vibratome for free-floating IHC. After peroxidase inactivation, sections were incubated in primary rabbit anti-GFP antibody (1:5000; Cell Signaling, RRID:AB_10692764) followed by Cy3-conjugated anti-rabbit secondary antibody (1:1000; Millipore, RRID:AB_92489) and counterstained using DAPI. Alternate sections were used to confirm placement of electrodes and optical implants. Viral expression was confirmed if GFP label was seen in both the MDT and in mPFC terminals. It is important to note that viral spread and uptake could not be precisely localized in MDT six to seven weeks after injection, and likely included thalamic regions adjacent to the MDT. Nonetheless, any terminals labeled in the mPFC, and thus activated optically, arose from the thalamic afferent. Rats would be eliminated if GFP expression was not observed in terminals in the mPFC. 2 rats were eliminated due to these criteria. These rats were in the NS-LTD and CUS-LTP groups.

#### Experiment 3: pathway specificity of optogenetically-induced plasticity

To test that plasticity induced by stimulation of thalamic terminals in the mPFC was pathway-specific, in an additional group of eight rats (females, *n* = 4; males, *n* = 4) that had been injected with virus in the MDT, responses evoked by stimulation of a separate afferent pathway from the ventral hippocampus were recorded in the mPFC. A bipolar stainless-steel stimulating electrode was lowered into the right ventral hippocampus (from bregma; AP: −6.0 mm, ML: +5.4 mm, DV –7.5 mm), and a recording electrode paired with an optic fiber was placed in the ipsilateral mPFC (from bregma; AP: −3.0 mm, ML: +0.6 mm, DV –3.5 ± 0.5 mm). Field potentials evoked in the mPFC by stimulation of the ventral hippocampal afferent were recorded as above, followed by optogenetic stimulation of the thalamic terminals in the mPFC using either the LTD or LTP protocols. Immediately after induction, electrical stimulation of the ventral hippocampus was resumed and responses were recorded for 2 h. Baseline was calculated and changes in response after optical stimulation were analyzed as above. After the experiment, brains were collected to confirm placement of electrodes and GFP expression. No rats were removed from this experiment for misplacement of electrodes. Further, any case in which GFP expression was not observed in mPFC terminals would be eliminated, and no rats were removed for this reason. Finally, no GFP expression was ever observed in the ventral hippocampus.

#### Experiment 4: cognitive set-shifting after LTD induction in the thalamic-mPFC pathway

To explore the relationship between reduced responsiveness of the mPFC to excitatory thalamic afferent activation and changes in cognitive flexibility, we induced opto-LTD before the set-shifting task in the AST. Optical probes were surgically implanted above the mPFC (from bregma; AP: −3.0 mm, ML: +0.6 mm, DV –3.5 ± 0.5 mm) during viral injection surgeries to allow laser stimulation of thalamic afferent terminals in the mPFC in awake, behaving rats. Six weeks after viral injection, 36 rats (females, *n* = 13; males, *n* = 23) underwent the AST test as described above. Before the ED task, rats were removed from the arena and either underwent LTD induction as described above, or were attached to the laser but received no stimulation. After induction or attachment to the laser, all animals were placed in their home cage for 1 h to allow full expression of LTD, and then returned to the AST arena to complete testing on the ED task. A separate control group received LTD induction before the ID task to ensure that overall performance capability was not affected non-specifically by LTD.

#### Experiment 5: rescue of CUS-induced set-shifting deficits by opto-LTP induction

As the duration of CUS treatment differed for males and females (Table 1), CUS began either three weeks (females) or four weeks (males) after viral injection and probe implantation as described in experiment 4. Unstressed groups received daily handling. A total of 45 rats (females, *n* = 26; males, *n* = 19) underwent AST testing as described above. Before the ED task, rats were removed from the arena and either received opto-LTP induction, or were attached to the optical cable but received no laser stimulation. After opto-LTP induction or attachment, all animals were placed back in their home cage for 30 min to allow full expression of LTP. Rats were then returned to the AST arena to complete testing on the ED set-shifting task. Non-stressed control rats that received no laser stimulation then underwent a second day of AST testing with the order of cues switched (odor to medium/medium to odor). On this second test sequence, they received LTP induction before the second reversal task (R2) to test the alternative interpretation that LTP induction may have erased previous learning, which could also account for an apparent improvement in set-shifting.

### Statistical analysis

Behavioral data were analyzed by one-way or two-way ANOVA (e.g., stress × optical stimulation) using GraphPad Prism (version 6.00 for Mac, GraphPad Software). Pairwise comparisons to detect specific group differences were performed using the Newman–Keuls test. Evoked potentials were analyzed by two-way repeated measures ANOVA with multiple comparisons by the Tukey test. Significance was determined at *p* < 0.05. Both sexes were included in all experiments. While these experiments were not powered to test sex differences, sex is included as a factor in the analyses, and as per NIH requirement, data are shown for both sexes separately.

## Results

### Experiment 1: validation of CUS-induced set-shifting deficits in female rats

**Figure 1.**
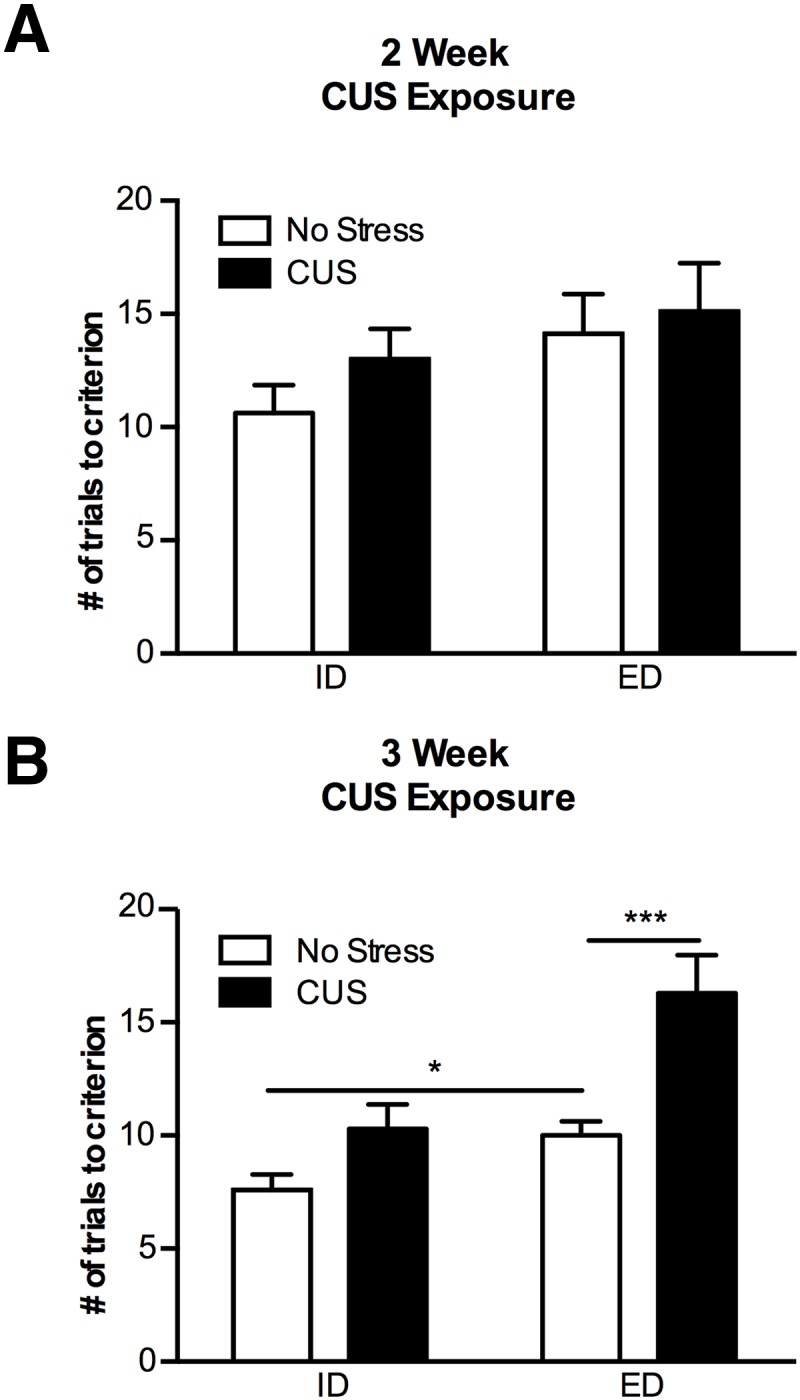
Females exhibit impaired set-shifting after 21 d of CUS, but not after 14 d of CUS. ***A***, In female rats receiving 14 d of CUS, performance on the set-shifting task was not different from unstressed controls (*p* = 0.73; *n* = 8–9)**. *B***, In a separate cohort of female rats, a modified 21-d CUS protocol induced a set-shifting deficit comparable to that seen in males after 14 d of CUS (*p < 0.05, **p < 0.01, ***p < 0.001, *p* = 0.0001; *n* = 5–6). Formation of a cognitive set was also demonstrated by a significant difference between the number of trials for the ID versus ED shifts (*p* = 0.03; *n* = 5–6).

Before the use of females in CUS experiments, we characterized the effects of CUS on ED set-shifting in female rats. Females exposed to the standard 14-d CUS protocol used for males (Table 1) did not exhibit differences in set-shifting compared to unstressed controls (*t*_(15)_ = 0.35; *p* = 0.73; *n* = 8–9; Fig. 1*A*)_a_ (Table 2). However, 21-d CUS impaired set-shifting in females compared to controls (*t*_(9)_ = 6.949; *p =* 0.0001; *n* = 5–6/group; Fig. 1*B*)_b_, similar to deficits observed after 14-d CUS in males (Bondi et al., 2008). Nonstressed females formed a cognitive set, as the ED shift required significantly more trials than the ID shift (*t*_(8)_ = 2.589; *p* = 0.03; *n* = 5–6/group; Fig. 1*B*)_c_. By contrast with females, male rats failed to complete AST testing after 21-d CUS treatment (data not shown). Therefore, as our goal was to produce similar CUS-induced deficits on set-shifting in males and females, a 14-d CUS treatment was used for males, and a 21-d CUS treatment was used for females in subsequent experiments.

**Table 2.** Statistical table

	Data structure	Type of test	Power
a	Real number Ratio scale, normal distribution	Student’s *t* test	95% CI [–4.980, 6.952]
b	Real number Ratio scale, normal distribution	Student’s *t* test	95% CI [–9.721, –4.946]
c	Real number Ratio scale, normal distribution	Student’s *t* test	95% CI [0.2615, 4.539]
d	Real number Ratio scale, normal distribution	Two-way ANOVA	NS-ChETA [56.58, 71.57] NS-GFP [96.70, 103.4] CUS-ChETA [88.29, 92.50]
e	Real number Ratio scale, normal distribution	Two-way ANOVA	NS-ChETA [122.2, 137.7] NS-GFP [92.08, 96.81] CUS-ChETA [106.5, 113.2]
f	Real number Ratio scale, normal distribution	Two-way ANOVA	LTD [94.99, 99.63] LTP [87.84, 92.85]
g	Real number Ratio scale, normal distribution	Two-way ANOVA	ChETA-no laser [8.015, 13.84] GFP-LTD [10.05, 15.75] ChETA-LTD [15.55, 27.52]
h	Real number Ratio scale, normal distribution (except where *n* are too small, e.g., by sex)	One-way ANOVA	ChETA-no laser [6.55, 15.89] GFP-LTD [10.70, 18.63] ChETA-LTD [13.74, 33.76]
i	Real number Ratio scale, normal distribution (except where *n* are too small, e.g., by sex)	One-way ANOVA	ChETA-no laser [6.823, 13.98] GFP-LTD [5.68, 14.82] ChETA-LTD [12.73, 23.27]
j	Real number Ratio scale, normal distribution	Two-way ANOVA	ChETA-no laser [7.337, 14.18] GFP-LTD [5.68, 14.82] ChETA-LTD [8.393, 12.75]
k	Real number Ratio scale, normal distribution	One-way ANOVA	ChETA-no laser [7.029, 10.54] GFP-LTD [5.436, 14.56] ChETA-LTD prior to ID [4.346, 9.939]
l	Real number Ratio scale, normal distribution	One-way ANOVA	NS-no laser [9.438, 15.16] NS-LTP [7.346, 12.84] CUS-GFP-laser [12.98, 17.82] CUS-ChETA-no laser [11.41, 16.90] CUS-ChETA-LTP [5.046, 9.240]
m	Real number Ratio scale, normal distribution	Two-way ANOVA	Male [7.596, 15.94] Female [7.531, 14.86]
n	Real number Ratio scale, normal distribution	Two-way ANOVA	NS-no laser [7.842, 13.04] NS-LTP [7.64, 13.01] CUS-GFP-laser [7.525, 15.28] CUS-ChETA-no laser [7.60, 14.15] CUS-ChETA-LTP [7.373, 15.63]
o	Real number Ratio scale, normal distribution	Student’s *t* test	95% CI [–6.727, 2.727]

### Experiment 2: validation of optogenetically-induced plasticity

#### Demonstration of LTD induction

**Figure 2.**
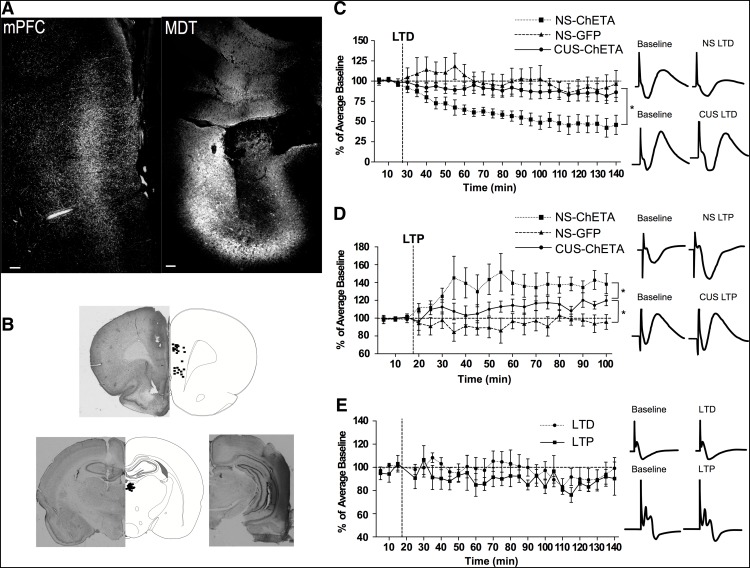
Validation of viral expression and optical induction of plasticity. Confirmation of electrode placement for both LTD and LTP recording experiments. ***A***, Representative image of GFP-immunofluorescence in the mPFC from an animal injected with AAV5-CaMKIIα-ChETA-YFP into the MDT six weeks previously. Shown are GFP+ thalamic afferent terminals in the mPFC (left), and the injection site in the MDT (right), verifying that ChETA was expressed in terminals receiving light stimulation (scale bar = 100 μm). ***B***, Recording electrode placement in the mPFC (top) and stimulating electrodes in the MDT (bottom left) in experiment 2. Bottom right micrograph shows a representative example of electrode placement in the ventral hippocampus in experiment 3. ***C***, Induction of opto-LTD in the mPFC of anesthetized rats six weeks after viral injection. Opto-LTD stimulation in non-stressed rats reduced the thalamic afferent-evoked response to 55.4 ± 2.5% of baseline (mean ± SEM; *p < 0.05). Control rats microinjected with AAV5-CaMKIIα-GFP, thus lacking ChETA expression, showed no change in response after the LTD protocol (98.6 ± 2.7% of baseline). Rats that had undergone CUS treatment also did not exhibit a reduction in field potentials after the opto-LTD protocol (88.2 ± 1.3% of baseline), suggesting that the reduced response reported previously following CUS occluded the induction of LTD. ***D***, Induction of opto-LTP in the mPFC of anesthetized rats six weeks after viral injection. After opto-LTP stimulation in nonstressed controls, evoked responses averaged 135.4 ± 4.0% of baseline (mean ± SEM; *p < 0.05) for at least 3 h after stimulation. Opto-LTP stimulation in control animals microinjected with AAV5-CaMKIIα-GFP six weeks prior, thus lacking ChETA channels, induced no change in response over the same 3-h period (98.0 ± 2.4%). Opto-LTP stimulation also significantly increased the thalamic afferent-evoked responses in the mPFC of CUS-treated rats to 111.6 ± 2.9% of baseline, although the potentiation was less than in nonstressed rats (*p* < 0.01). ***E***, Field potentials recorded in the mPFC in response to stimulation of the ventral hippocampus were not changed by optical stimulation of thalamic afferent terminals in the mPFC (*p* = 0.30).

To demonstrate that low frequency light stimulation of ChETA-expressing thalamic afferent terminals in the mPFC depressed the evoked response, we conducted *in vivo* recordings of field potentials evoked in the mPFC by electrical stimulation in the MDT of anesthetized rats (Fig. 2*A*,*B*). After opto-LTD induction, evoked field potential magnitude averaged 55.4 ± 2.5% of baseline (mean ± SEM) after reaching a stable level of depression ∼1 h after induction, which persisted for the duration of recording (main effect for treatment: *F*_(2,9)_ = 5.944; *p* = 0.02; main effect for time; *F*_(25,225)_ = 9.370; *p* = 0.0002; time × treatment interaction: *F*_(50,225)_ = 3.099; *p* < 0.0001; Fig. 2*C*)_d_. Laser stimulation in GFP controls lacking ChETA expression produced no change in evoked responses (98.6 ± 2.7% of baseline over the same time-frame), ruling out the possibility that non-specific effects of laser stimulation caused the depressed response. A separate group of rats was used to test for occlusion of LTD after CUS. Rats that underwent CUS did not exhibit a significant reduction in field potential response after the opto-LTD protocol (88.2 ± 1.3%), suggesting that CUS occluded the induction of opto-LTD (Fig. 2*C*).

#### Demonstration of LTP induction

In another cohort of rats, high-frequency light stimulation of ChETA-expressing thalamic afferent terminals in the mPFC significantly potentiated the evoked response (main effect of treatment: *F*_(2,10)_ = 4.804, *p* = 0.03; main effect for time: *F*_(19,190)_ = 2.473; *p* = 0.001; time × treatment interaction: *F*_(38,190)_ = 1.815, *p* = 0.01; *n* = 4–6/group; Fig. 2*D*)_e_. After high-frequency light stimulation in nonstressed rats, responses were 135.4 ± 4.0% of baseline, and potentiation was maintained for the duration of the recording period. High-frequency stimulation in GFP-control rats lacking ChETA channel expression produced no change in evoked responses (98.0 ± 2.4%). Interestingly, the potentiation of evoked response after high-frequency laser stimulation was slightly lower in CUS-treated animals (111.6 ± 2.9%), which were different from both the nonstressed rats showing opto-LTP (*p* < 0.01) and GFP control rats receiving laser stimulation (*p* < 0.01). Nonetheless, the magnitude of potentiation of the response even in the stressed rats was consistent with that seen after electrical induction of LTP in the MDT-mPFC pathway (Herry and Garcia, 2002). Together, the results of experiment 2 demonstrate opto-LTP induction after laser stimulation of ChETA-expressing thalamic afferent terminals in the mPFC of both stressed and unstressed animals, and opto-LTD induction in unstressed animals that appears to be occluded by CUS.

### Experiment 3: pathway specificity of optogenetically-induced plasticity in the mPFC

To confirm that plasticity induced by optical stimulation of the thalamic afferent terminals in the mPFC was specific to the thalamic-mPFC pathway and did not affect other mPFC afferent pathways, a separate cohort of rats received viral injection of ChETA in the MDT, and responses evoked in the mPFC by stimulation from a different afferent, the ventral hippocampus were recorded. Field potentials evoked in the mPFC by stimulating in the ventral hippocampus were unchanged from baseline after thalamic afferent terminal stimulation with either the opto-LTD or opto-LTP protocol (main effect of treatment: *F*_(1,6)_ = 1.269, *p* = 0.30; interaction: *F*_(26,156)_ = 0.415, *p* = 0.99 *n* = 3–5/group; Fig. 2*E*)_f_. This indicates that optogenetic plasticity induced by stimulation of thalamic afferent terminals in the mPFC does not alter the response to other afferent inputs.

### Experiment 4: attentional set-shifting after LTD induction in the thalamic-mPFC pathway

**Figure 3.**
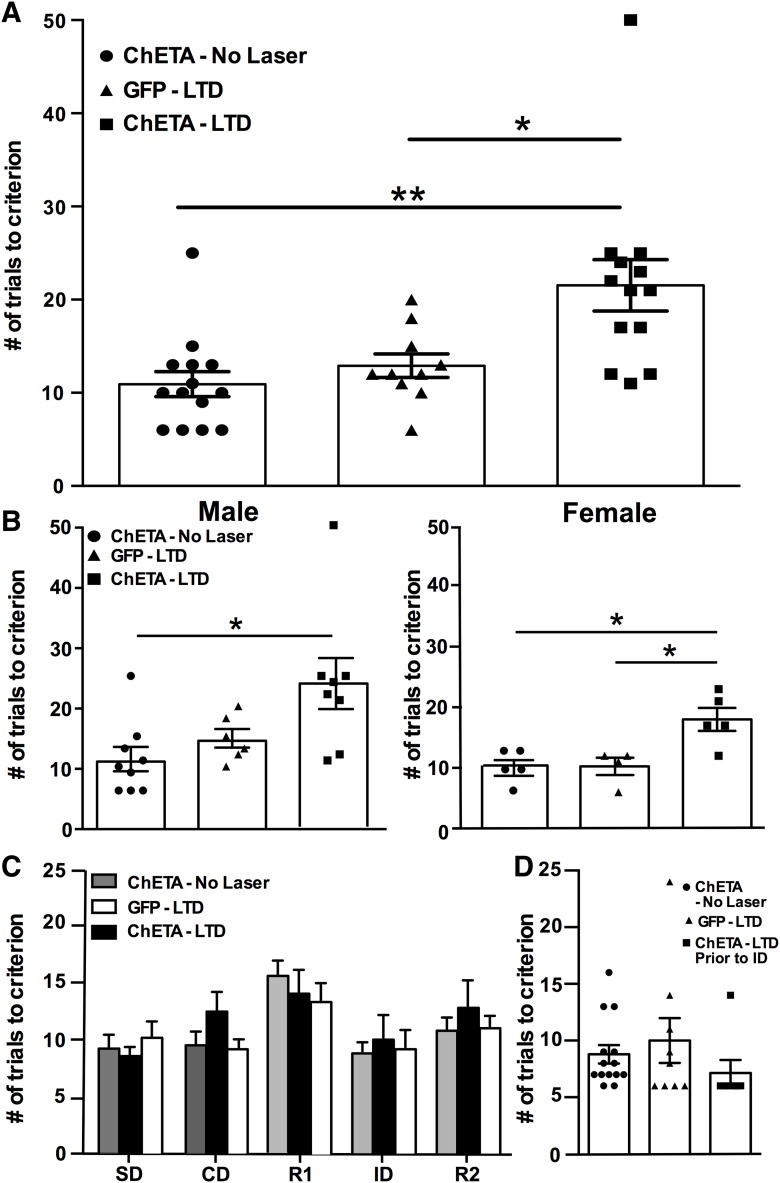
Induction of opto-LTD results in deficits in ED set-shifting. ***A***, Opto-LTD was induced immediately after the R2 task, and rats were allowed 1 h in the home cage for LTD induction before testing on the ED task. Rats expressing ChETA and receiving opto-LTD stimulation required significantly more trials to reach criterion on the ED set-shifting task than controls (*p < 0.05; **p < 0.01). ***B***, Opto-LTD effects reported by sex. Both male (left; *n* = 6–9/group) and female (right; *n* = 4–5/group) rats exhibited deficits in set-shifing after opto-LTD (*p < 0.05). ***C***, Before LTD induction, trials to criterion on the SD, CD, and R1 tasks were similar between groups, indicating no baseline differences before laser treatment (*p* = 0.71). ***D***, Opto-LTD induction before the ID shift task had no effect on performance, demonstrating that LTD in the thalamic-mPFC pathway did not induce non-specific deficits in overall learning or task performance capability (*p* = 0.40).

Rats were tested on the AST six weeks after viral injections and probe implantation. Immediately after completion of the R2 task, opto-LTD was induced as described above. Non-LTD control groups were attached to the laser but received no stimulation and all rats were allowed 1 h in the home cage for full LTD induction. They were then returned to the testing arena to complete the ED set-shifting task. On the set-shifting task following laser stimulation, ChETA-LTD rats required significantly more trials to reach criterion than either GFP controls or ChETA-expressing animals that did not receive laser stimulation (two-way ANOVA; effect of treatment *F*_(2,31)_ = 7.350; *p* = 0.002; with no effect of sex *F*_(1,31)_ = 2.370; *p* = 0.13; *n* = 10–13/group total; male = 6–9/group; female = 4–5/group; Fig. 3*A*)_g_. Opto-LTD induced deficits in both male (one-way ANOVA; *F*_(2,20)_ = 5.045; *p* = 0.017; *n* = 6–9/group)_h_ and female rats (one-way ANOVA; *F*_(2,11)_ = 7.998; *p* = 0.007; *n* = 4–5/group; Fig. 3*B*)_i_. Performance of all groups were similar on the tasks preceding LTD induction, i.e., on the SD, CD, reversal learning (R1 and R2), and the ID shift tasks (two-way ANOVA; *F*_(8,165)_ = 0.683; *p* = 0.71; Fig. 3*C*)_j_. Further, there was no effect on performance on the ID shift task in the control group that received LTD induction before that task (one-way ANOVA; *F*_(2,27)_ = 0.95; *p* = 0.40; *n* = 7–14/group; Fig. 3*D*)_k_, demonstrating that inducing LTD in the mPFC did not non-specifically decrease overall learning or performance capability.

### Experiment 5: rescue of CUS-induced set-shifting deficits by opto-LTP induction

**Figure 4.**
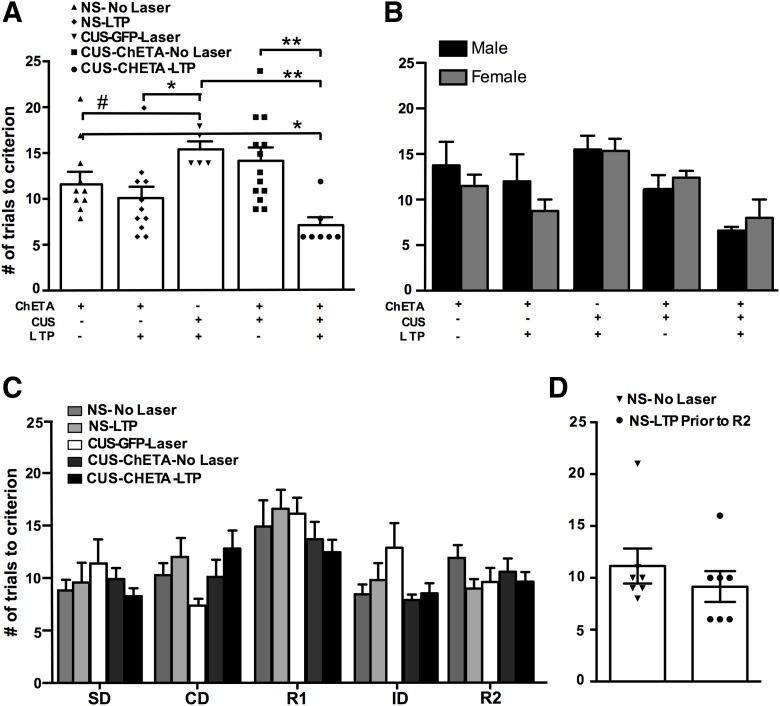
Induction of LTP in the thalamic-mPFC pathway rescued CUS-induced deficits in set-shifting. ***A***, CUS-treated animals receiving opto-LTP required significantly fewer trials to reach criterion on the set-shifting task than both the CUS-GFP and CUS-ChETA no-laser control groups, as well as the nonstress (NS) no-laser control group (*p < 0.05, **p < 0.01, ***p < 0.001, #*p* = 0.07; *n* = 5–13/group) ***B***, Set-shifting groups reported by sex. There was no difference between males and females in any treatment group. ***C***, Before LTP induction, SD, CD, and R1 were similar between groups, indicating no baseline differences before laser treatment (*p* = 0.11). ***D***, Induction of LTP in the mPFC before the R2 had no effect, suggesting that LTP does not non-specifically alter the previously learned cognitive set (*p* = 0.47).

To test whether potentiating responsivity in the thalamic-mPFC pathway could rescue set-shifting that had been compromised by CUS, we induced opto-LTP in CUS-treated animals before the set-shifting task. Both CUS and nonstressed groups that received no laser stimulation exhibited higher number of trials in the ED stage compared to the ID stage (*t* test; *p* < 0.004; data not shown), indicating that the animals had acquired a cognitive set. Opto-LTP facilitated performance on the ED set-shifting task following stress (one-way ANOVA; *F*_(4,41)_ = 5.48; *p* = 0.001; *n* = 5–13/group; Fig. 4*A*)_l_. ChETA-expressing animals that received both CUS and opto-LTP required fewer trials to complete the set-shifting task than either CUS-treated GFP-expressing controls (*p* < 0.001) or CUS-treated ChETA-expressing controls that received no laser stimulation (*p* < 0.01). Additionally, while CUS-treated rats receiving opto-LTP had a similar number of trials to criterion as nonstressed rats receiving LTP, the CUS-LTP rats required fewer trials than nonstressed controls that did not receive LTP (*p* < 0.05). There was no difference between male and female rats in any treatment group (two-way ANOVA *F*_(4,30)_ = 0.8232; *p* = 0.521; Fig. 4*B*)_m_. There were no baseline differences between groups in the tasks preceding the ED task that could have accounted for effects seen after the induction of opto-LTP (two-way ANOVA; *F*_(16,200)_ = 1.444; *p* = 0.124; Fig. 4*C*)_n_.

There was also no difference between animals receiving LTP induction before R2 and controls (*t*_(12)_ = 0.9218, *p* = 0.37, *n* = 7/group; Fig. 4*D*)_o_, indicating that previous learning on the AST was not disrupted by opto-LTP induction. Thus, the results of this experiment suggest that potentiating responses evoked in the mPFC by activation of the thalamic afferent can correct CUS-induced set-shifting deficits.

## Discussion

The results of these studies demonstrate the use of opto-LTD and opto-LTP as viable tools for inducing bidirectional pathway-specific plasticity in the mPFC. More specifically, the implementation of these approaches in the current studies indicate a beneficial effect of potentiating the thalamic-mPFC pathway in rescuing stress-induced deficits in cognitive flexibility, and directly implicate reduced responsivity in the thalamic-mPFC pathway in producing set-shifting deficits. To induce opto-LTD or opto-LTP, the ChETA variant of channelrhodopsin was chosen specifically for its rapid kinetics (Gunaydin et al., 2010). The ChETA channel is capable of following at a higher stimulation frequency than wild-type channelrhodopsin, with less extra spiking and reduced spike variability, allowing for more reliable stimulation at the high-frequency rates needed to induce LTP, without the inactivation seen using previous variants (Gunaydin et al., 2010). The ability to induce either opto-LTD or opto-LTP *in vivo* using the same channel by varying the stimulation pattern and frequency also allows for less variability in pathway-specific studies, and simplifies the design of such experiments.

Using this approach in the present studies, we describe the effects of inducing plastic changes in the thalamic-mPFC pathway on mPFC-dependent set-shifting. We report that induction of opto-LTD through stimulation of thalamic afferent terminals within the mPFC results in deficits in ED set-shifting similar to those observed after CUS, without affecting overall learning or task performance capabilities. This implies a direct relationship between decreased responsivity observed previously in the mPFC after chronic stress and the associated deficits in cognitive flexibility. Chronic stress disrupts the function of the mPFC at multiple levels, decreasing the response to afferent stimulation and attenuating acute stress-evoked increases in extracellular glutamate levels (Jett et al., 2017). Chronic stress also decreases glutamate receptor expression in the mPFC (Yuen et al., 2012), and causes neuroanatomical changes including hypertrophy of local inhibitory interneurons (Gilabert-Juan et al., 2013), and reduced dendritic complexity of pyramidal neurons in the mPFC (Liston et al., 2006). Our results suggest that rather than hypoactivity per se, stress-induced cognitive deficits may be related to hypo-responsivity of the mPFC to specific thalamic afferent input.

Furthermore, our data and others suggest that such effects may be due to alterations in specific afferent pathways. CUS decreased local field potentials evoked in the mPFC by stimulation of the thalamic afferent, but not the ventral hippocampus (Jett et al., 2017). Likewise, the NMDA receptor subunit GluN2B specifically regulates synaptic input from the MDT, through which activation can attenuate depressive-like behaviors, suggesting pathway-specific molecular differences as well (Miller et al., 2017). Further evidence that chronic stress can disrupt specific prefrontal circuits mediating higher order executive processes was reported in a recent study showing that chronic stress produced a functional disconnect in a prefrontal-striatal pathway that specifically targets striosomes, resulting in disruption of cost-benefit conflict decision making (Friedman et al., 2017). Such observations, together with the data presented in the present paper, highlight the importance of investigating pathway specific mechanisms in the mPFC, rather than simply focusing on overall activity of the mPFC after stress. The ability to induce LTD or LTP in specific afferent pathways using optogenetics allows such investigations. However, an important limitation of the approach used in the present study, specifically measuring changes in afferent-evoked local field potentials, is that we cannot determine if the changes in response seen following either stress or optogenetic stimulation were the result of a change in synaptic strength or a change in the number of cells activated. More refined electrophysiological approaches, e.g., patch clamp recordings, would be necessary to address this question.

CUS resulted in a reduction in opto-LTP induction compared to nonstressed controls, although the potentiation induced in stressed rats was still capable of rescuing set-shifting behavior. Other studies have reported a reduction in electrical LTP in the mPFC after exposure to stress (Goldwater et al., 2009; Schayek and Maroun, 2015; Khazen et al., 2018). Interestingly, the rescue of set-shifting by opto-LTP in the thalamic-mPFC pathway appears to be specific, as promoting LTP in the hippocampal-mPFC pathway by acute injection of MK-801 resulted in deficits in set-shifting in nonstressed animals (Blot et al., 2015).

In the hippocampus, LTP has been associated with memory consolidation (Howland and Wang, 2008). However, in the present experiments, LTP in the thalamic-mPFC pathway did not appear to comprise a memory consolidation process, but rather to be more involved in the enhancement of performance on the set-shifting task. If a major role of the mPFC in set-shifting is inhibition of inappropriate responses (Hauser, 1999; Bissonette et al., 2013), then facilitating that process by strengthening specific afferent inputs to the mPFC may improve cognitive capabilities that depend on such inhibition (Ragozzino, 2007). Thus, it is possible that LTP induced in the thalamic-mPFC afferent pathway improved performance by promoting more effective inhibition of downstream circuits maintaining responses that are no longer effective (Herry and Garcia, 2002). This possibility is consistent with the observation that high-frequency stimulation in the IL mPFC also facilitated performance on an extinction learning task (Maroun et al., 2012).

It is often suggested that females are more sensitive to stress than males (Sheikh et al., 2002; Tolin and Foa, 2006). However, several convergent animal studies addressing sex differences in the response to stress have indicated that females can show more resilience than males to the same chronic stress procedures, including those that cause PFC dysfunction (Luine et al., 2007; Wei et al., 2014). In the present study, a modified 21-d CUS paradigm, but not 14-d CUS, impaired performance in the mPFC-mediated set-shifting task in females comparably to the stress-induced set-shifting deficits reported in male rats after 14-d CUS (Bondi et al., 2008; Jett et al., 2017; Fucich et al., 2018). It is possible that removing social defeat stress from the female stress protocol contributed to the need for longer CUS treatment to reach the same behavioral deficit, as social defeat is one of the more robust stress procedures in the CUS protocol. However, males exposed to the same 21-d CUS paradigm as females would not complete AST testing, indicating that this paradigm was nonetheless more severe for males than it was for females. Thus, rather than treating both sexes for the same period of time, which would be necessary, for example, to explicitly study sex differences in stress vulnerability, our goal for this study was instead to achieve a common endpoint, i.e., to induce a comparable deficit in set-shifting in both male and female rats. Thus, the use of an extended CUS procedure in females allowed for the inclusion of both sexes to investigate mechanisms of stress-induced cognitive dysfunction in the mPFC and its rescue by optogenetically-induced plasticity. Our results showed that optogenetically depressing activity in the thalamic-mPFC pathway had similar detrimental effects on cognitive set-shifting in both sexes. Likewise, we showed that optogenetically potentiating responsivity in the thalamic-mPFC pathway had similar beneficial effects in both sexes on cognitive set-shifting that had been comparably compromised by stress.

Induction of opto-LTD within thalamic afferent specific terminals in the mPFC compromised set-shifting, and opto-LTP corrected set-shifting deficits induced by CUS. This bidirectional control of set-shifting behavior further supports the role of plasticity in the thalamic-mPFC pathway in cognitive flexibility. Furthermore, opto-LTD was occluded in stressed animals, indicating that the thalamic-mPFC pathway was already suppressed, possibly by a similar mechanism, following CUS treatment. This finding has potential translational relevance, as it has been reported previously that extinction learning in stressed rats, as a model of behavioral therapy, restored thalamic-evoked field potential responses in the mPFC and reversed the CUS-induced set-shifting deficit (Fucich et al., 2018). Further, preventing protein synthesis in the ventral mPFC, which is required for LTP (Nguyen et al., 1994), blocked the therapeutic effects of extinction learning (Fucich et al., 2018). Based on the data we have presented here, it is likely that extinction learning after CUS induced plasticity that specifically strengthened the thalamic-mPFC pathway, resulting in the rescue of set-shifting behavior.

An alternative interpretation might be that, rather than facilitating set-shifting after stress, opto-LTP stimulation in the mPFC instead ablated the prior learning that occurred on the preceding tasks, e.g., formation of a cognitive set, thus reducing the difficulty of shifting to the other dimension in the set-shifting task. To test for this possibility, a separate group of rats received opto-LTP stimulation before the R2 task, in which we typically see improvement compared to the first reversal task, indicative of prior learning. Thus, if previous learning had been erased by laser stimulation, the number of trials to criterion on R2 would have increased compared to controls. However, there was no difference between animals receiving LTP induction before R2 and controls, indicating that previous learning on the AST was not disrupted by opto-LTP induction. Thus, the results of this experiment suggest that potentiating responses evoked in the mPFC by activation of the thalamic afferent can correct CUS-induced set-shifting deficits.

Several important controls were included in the present set of experiments. First, stimulating thalamic afferent terminals in the mPFC with parameters that induced opto-LTD or opto-LTP in that afferent pathway did not change field potentials evoked by stimulating the ventral hippocampus, indicating that responses to other afferent pathways were unaffected by optogenetic stimulation of the thalamic-mPFC pathway. Behavioral control groups in the present study addressed other potential confounds. Opto-LTD stimulation did not induce deficits in the ID task, indicating that animals could perform normally on tasks for which the mPFC is not required. Additionally, GFP-expressing animals exhibited set-shifting behavior comparable to that of no-laser controls, suggesting that potential non-specific effects of laser stimulation per se were not responsible for the changes in set-shifting. Another possible concern was that opto-LTP may have erased the prior learning that established the cognitive set in the sequence of tasks preceding stimulation. This was not the case, as LTP did not change the number of trials to criterion in the R2 task, which is also affected by prior learning. Interestingly, CUS-treated animals receiving opto-LTP stimulation required significantly fewer trials to reach criterion than non-stressed, non-optically stimulated control rats. It is possible that this improvement in set-shifting in stressed rats relative to controls was due to the emergence of positive compensatory processes that had developed in response to chronic stress, but which only became apparent when the detrimental effect of CUS was reduced by LTP. Further studies exploring molecular and anatomic changes induced in the mPFC of stressed animals may reveal more information regarding the mechanisms underlying such potential compensatory mechanisms.

One drawback of this study is that the mPFC is treated as a whole, rather than separating out the prelimbic (PrL) and IL subregions of the mPFC. Both IL and PrL regions are susceptible to the effects of chronic stress, with both subregions expressing decreased dendritic complexity after stress (Cook and Wellman, 2004; Goldwater et al., 2009). Further, optogenetically activating either the IL or PrL produces antidepressant effects (Covington et al., 2010; Fuchikami et al., 2015). These commonalities may be through shared afferents. Both the PrL and IL receive input from the thalamic afferent and several other regions, such as the hippocampus and amygdala, but also have their own unique inputs (Vertes, 2004). Likewise, altering plasticity within both subregions can affect different downstream targets, resulting in different outcomes (Hoover and Vertes, 2007). For example, chronic stress impairs IL-dependent behaviors, such as fear extinction learning and retrieval (Farrell et al., 2010) while stress enhances the expression of conditioned fear, a PrL-dependent behavior (Farrell et al., 2013). More targeted studies in the future may further parse out the specific roles of these mPFC subregions in set-shifting.

Finally, there is potential translational relevance to the current results, as they suggest that interventions that increase plasticity in the response of mPFC to afferent input may be beneficial in reducing deficits in cognitive flexibility that are frequently present in stress-related anxiety and mood disorders such as PTSD or depression. The induction of opto-LTD and opto-LTP using the ChETA variant of channelrhodopsin offers a viable approach with which to investigate such mechanisms further.

## Synthesis

Reviewing Editor: Carmen Sandi, Swiss Federal Institute of Technology Decisions are customarily a result of the Reviewing Editor and the peer reviewers coming together and discussing their recommendations until a consensus is reached. When revisions are invited, a fact-based synthesis statement explaining their decision and outlining what is needed to prepare a revision will be listed below. The following reviewer(s) agreed to reveal their identity: Elena Vazey.

This study provides support for a specific thalamic-prefrontal pathway deficit that accounts for some of the cognitive deficits resulting from chronic stress exposure. Prefrontal cognitive deficits following stress have been previously attributed to be due to generalized hypoactivity. These data substantially redirect thinking about this matter. In addition, the data show novel observations that induction of LTP-like changes in the same pathway enhances acquisition of learning. The two reviewers and editor are enthusiastic about the study but have minor concerns about the quality and rigor of the data reporting, and the basis for some of the parameters used in the study. The article would benefit from several additional clarifications, and revisions to figures, methods and statistical analyses detailed below:

Anatomical specificity:

In the Introduction section, the authors mention that MDT input to mPFC is predominantly to neurons in layer 2/3. Yet, in Fig. 2B, their data clearly show that innervation is mostly focused in the deepest layer 6, with some nominal labeling in layer 1, and is virtually absent in layer 2/3. This, coupled with the lack of clarity about where in the MDT injection site image cells took up virus (i.e., since the opsin-GFP fusion proteins are preferentially expressed on axons, not soma, this complicates distinguishing the exact site of injection), raises questions about whether the authors are studying the pathway that they claim to be. Several other midline thalamic nuclei are known to innervate deep-layer, and not superficial, aspects of mPFC. Considering that injection volumes were 0.5 ul/ side, and injections were only placed 0.9 mm lateral to the midline, suggests that viral spread medially could have encompassed adjacent-lying and midline aspects of the thalamus. From a conceptual standpoint, the data are what they are, so the study isn't necessarily weakened by exactly where in fact the thalamic inputs arise from. Nevertheless, more careful accounting may improve the overall quality of the reporting. If the authors are not sure, then they should consider relaxing their definition from MDT to something more general (i.e., as they have done so appropriately in their explanation of mPFC subregion).

It would be important to provide some depiction of the GFP expression profile or especially the hippocampal implant locations in the ventral hippocampal recording during MDT-mPFC pathway manipulations. In addition, it would be important to display some examples of evoked activity in the ventral hippocampal-mPFC pathway.

Presentation of evoked activity data:

There is an extensive amount of data that has been collected in this study involving evoked and field potential measurements that bidirectionally alter evoked activity. However, not much detail is provided in the presentation of data. Noteworthy absences are examples of representative field potentials and how they are changed by the optogenetic manipulations, both increasing and decreasing as a function of the respective LTP and LTD protocols. Such data are helpful in allowing the reader to gain a better appreciation of what the data look like, especially in terms of conceptualizing how these manipulations affect evoked activity in mPFC; they would also improve overall rigor in the data presentation.

Other technical issues:

-As the authors note in the Discussion section, ChETA is capable of responding to higher frequency stimulation than the wildtype form of channelrhodopsin. Yet, pyramidal neuron firing rates top out at around 40 Hz, and it is not clear what the significance is of stimulating terminals at such high frequency.

-In Figure 1B, is it the case that female rats subjected to the 3-week chronic stress manipulation show generalized impairments in intradimensional shift as compared with unstressed controls?

- Several instances of analyses requiring multifactor ANOVAs are currently analyzed as one way ANOVAs: It is not clear that the studies were appropriately powered for sex differences, but in cases when comparing both sexes eg Experiment 4, analyses should be done with sex as an additional factor in the ANOVA rather than multiple 1 way ANOVAs.

- Given the small size of the nucleus, histological inclusion criteria regarding electrode, fiber placement and viral spread within/from MDT are missing from the methods and need to be included. Additional details on the total number of trials performed before animals failed a stage and information about omission criterion should be given for the ASST.

- For all of the figures additional labeling for clarity would greatly aid understanding for the reader. In cases where axis labels are expected to apply to multiple panels, or comparisons between panels are highlighted axes need to scaled in parallel which is not currently the case.

- Did all groups return to their homecage for 75 minutes between IDR and ED, as was required for the animal undergoing LTD or LTP protocols?

Interpretive issues to consider in the Discussion:

1. An idea advanced by the field is that PFC hypoactivity is a feature of stress-related psychiatric disorders. The authors highlight the relevance of their data to inform this general idea (Discussion paragraph, lines 457-471). Nevertheless, it doesn't seem that the authors noted any explicit changes in baseline field potentials (or other neuronal activity) in the mPFC of rats following chronic stress exposure. Instead, the authors find that specifically manipulating the MDT-mPFC pathway is capable of overcoming cognitive deficits produced by stress. Thus, it seems like the data from this study challenges the general idea that PFC hypoactivity underlies stress-related cognitive dysfunction, and alternatively may be mediated via a deficiency in a specific pathway that might otherwise be latent.

Much evidence in the field has been advanced to suggest that LTP, or LTP-like processes, are critically important for the consolidation of memory. An interesting feature of this study is that the data suggest that LTP-like events are not a consolidation mechanism, but instead are more involved in the enhancement of task acquisition. Do these data challenge or change our thinking surrounding the role of LTP in memory formation? This would seem like a relevant point for the authors to discuss.

2. Although the authors have titrated the stressors to produce similar ASST deficits between sexes they incorrectly state that resident intruder paradigms are not relevant for stress studies in female subjects. Studies from other groups in recent years have effectively used lactating female residents in social defeat stress paradigms. As social defeat is only one of the stressors in the CUS paradigm this is not a particularly major concern. However, this does generate important considerations for the interpretation of Experiment 1 given how potent the physiological response is to social defeat paradigms. The lack of defeat stress rather may be a major contributing factor to the differential timecourse of stress exposure between males and females rather than a relative resistance suggested in Experiment 1, although variability in no stress groups may partially impact this conclusion. These factors should be included in the discussion.

3. Interpretation better suited for the discussion should be removed from the results for experiment 5 ~ line 425.

4. The purpose of Experiment 3 unclear. Optogenetic LTP and LTD protocols within the MDT-mPFC pathways are well established and validated. MDT-mPFC circuit changes are unlikely to impact plasticity in other circuits due to well established pre and postsynaptic components within a given path.

## Author Response

Response to Reviews for eNeuro ms #eN-NWR-0363-19

Note - all edits are identified in the marked version of the revised manuscript by red font

Comment 1: Please provide Table 1 in .doc file format.

Response. Table 1 has been provided as a .doc file

Comment 2: ...[considering] lack of clarity about where in the MDT injection site... cells took up virus... viral spread medially could have encompassed adjacent-lying and midline aspects of the thalamus. ...the study isn't weakened by where the thalamic inputs arise from. Nevertheless, more careful accounting may improve the overall quality of the reporting. If the authors are not sure, then they should consider relaxing their definition from MDT to something more general.

Response: The reviewers raise an excellent point. Injection sites were centered in the MDT, but other nearby thalamic areas may have been included in the spread of injection and viral uptake. We also agree with the reviewers that it is not essential to the data nor to our conclusions that the effect be localized solely to the MDT afferent. Therefore, we now raise this caveat in the methods on p. 7, and state that the injections were centered in the MDT, but we refer to the targeted pathway throughout the paper more generally as the “thalamic-mPFC afferent”.

Comment 3: It would be important to provide some depiction of the GFP expression profile or especially the hippocampal implant locations in the ventral hippocampal recording during MDT-mPFC pathway manipulations. In addition, it would be important to display some examples of evoked activity in the ventral hippocampal-mPFC pathway.

Response: To clarify, virus was never injected into the hippocampus, and we never observed GFP label in hippocampus after MDT injections. This is now stated at the end of the methods section for experiment 3. Also, we never recorded in hippocampus. In the test of pathway specificity in experiment 3, responses were evoked in mPFC by stimulation in ventral hippocampus, while plasticity was induced in the thalamic afferent.

To respond to the comment more generally, a new panel has been added to Figure 2 showing a representative example of stimulating electrode placement in the ventral hippocampus. Unfortunately, due to a technical error (summer SURF student), the majority of histological sections of ventral hippocampus were lost, so we are unable to provide a schematic portraying all electrode placements. But in all cases tested in this experiment, responses were successfully evoked in mPFC by hippocampal stimulation. To improve the clarity of this figure, panels A and B have been rearranged.

We also now state at the end of the methods sections for Experiments 2 and 3 that placement of electrodes were confirmed histologically, and that any rat in which the electrode placement was located outside the target region (MDT or mPFC) would be eliminated. We report the number of animals removed for misplacement of the stimulating electrodes outside the MDT in experiment 2. No animals were removed for misplacement of the recording electrodes in mPFC. We also state that animals in which no GFP expression was observed in mPFC terminals would be eliminated, and report the number of animals eliminated for this reason in experiment 2.

Comment 4: criteria regarding electrode, fiber placement and viral spread within/from MDT are missing from the methods and need to be included.

Response: In addition to the edits and additions described in the comment above, this is also now included at the end of the methods section for experiment 2.

Comment 5: It would be important to display some examples of representative evoked field potentials and how they are changed by the optogenetic manipulations,

Response: Figure 2 now includes representative examples of evoked field potentials recorded in the mPFC at baseline and after opto-LTD or opto-LTP stimulation of the thalamic afferent (Figs 2C-D) and ventral hippocampal afferent (Fig 2E).

Comment 6: As the authors note in the Discussion section, ChETA is capable of responding to higher frequency stimulation than the wildtype form of channelrhodopsin. Yet, pyramidal neuron firing rates top out at around 40 Hz, and it is not clear what the significance is of stimulating terminals at such high frequency.

Response: The high frequency optical stimulation is of the afferent terminals, not the pyramidal cells, the end result of which is potentiation of the evoked response to a single pulse. The purpose of the stimulation is not to drive firing of the pyramidal cells at the same rate, but to induce plasticity and potentiate their response to single-pulse stimulation of that afferent. The high rate of optical stimulation used in the present study to elicit potentiation is very similar to the rate of electrical stimulation used previously to potentiate that same pathway (reference cited in the manuscript).

Comment 7: In Figure 1B, is it the case that female rats subjected to the 3-week chronic stress manipulation show generalized impairments in intradimensional shift as compared with unstressed controls?

Response: The intradimensional shift was not consistently altered after stress in either males or females. In this particular experiment (Fig 1B), there was a slight increase (p=0.08), but this was not seen in other experiments (e.g., Fig 4C).

Comment 8: Several instances of analyses requiring multifactor ANOVAs are currently analyzed as one-way ANOVAs: It is not clear that the studies were appropriately powered for sex differences, but in cases when comparing both sexes eg Experiment 4, analyses should be done with sex as an additional factor in the ANOVA rather than multiple 1 way ANOVAs.

Response: a) The reviewer is correct that these studies were not powered for sex differences. But as per NIH requirements, after the primary analysis, we also report the data for each sex separately. We now state this more clearly in the statistics section of the methods.

b) Sex is now analyzed using multi-factorial ANOVA, and no significant differences were detected. Results are now reported as such in the statistical summary table and in the results sections for experiments 4 and 5. For both experiments, after conducting the primary analysis (i.e., multi-factorial ANOVA including sex as a factor), in showing the data separately by sex, we also still then analyze each alone by ANOVA.

Comment 9: Additional details on the total number of trials performed before animals failed a stage and information about omission criterion should be given for the ASST.

Response: The following has been added to the AST methods section:

“If no choice was made within the maximum trial time limit of 10 min, the trial was omitted and scored as a ‘no choice’ trial. If six ‘no choice’ trails occurred in a row (i.e. one hour without a dig), the rat was removed from the study.”

We now report how many animals were eliminated in each experiment for this reason, and from which groups.

Comment10: For all figures additional labeling for clarity would aid understanding. In cases where axis labels apply to multiple panels, or comparisons between panels are highlighted, axes need to be scaled in parallel.

Response: Additional axis labels have been added for clarity. Compared panels are now scaled in parallel.

Comment 11: Did all groups return to their homecage for 75 minutes between IDR and ED, as was required for the animal undergoing LTD or LTP protocols?

Response: Yes, all animals received equal wait times between ID and ED tasks. This has been clarified in the methods sections for Experiments 4 and 5, respectively.

Comment 12. ...it seems like the data from this study challenge the general idea that PFC hypoactivity underlies stress-related cognitive dysfunction, and alternatively may be mediated via a deficiency in a specific pathway that might otherwise be latent.

Response: We have addressed this point more directly, i.e., that our data seem to differentiate between hypoactivity of the mPFC and hypo-reactivity of the mPFC to specific afferent input, in the broader discussion in paragraph 2 of the discussion. It is also addressed in the broader discussion in paragraph 4 of the discussion.

Comment 13: the data suggest that LTP-like events are not a consolidation mechanism, but instead are more involved in the enhancement of task acquisition. Do these data challenge or change our thinking surrounding the role of LTP in memory formation? This would seem like a relevant point for the authors to discuss.

Response: This is now addressed in the new paragraph 5 of the discussion.

Comment 14: the authors... incorrectly state that resident intruder paradigms are not relevant for stress studies in female subjects... other groups have effectively used lactating female residents in social defeat... As social defeat is only one of the stressors in the CUS paradigm this is not a particularly major concern. However,... lack of defeat stress may be a contributing factor to the differential time course of stress exposure between males and females rather than a relative resistance suggested in Expt 1.

Response: a) In the Methods for experiment 1, we have revised the statement referenced above, as follows:

“Because of practical difficulties implementing social defeat stress in female rats (e.g., use of lactating females as residents), the social defeat stressors in the standard male protocol were replaced...”

b) In paragraph 6 of the discussion, we now consider the possibility that removal of social defeat stress from the CUS protocol may have contributed to the need for more days of treatment to achieve the same behavioral deficit. However, we also note that the same 21 protocol was too severe for males, suggesting a difference in vulnerability. Regardless, the end result was to apply different duration CUS for the same deficit.

Comment 15: Interpretation better suited for the discussion should be removed from the results for experiment 5 ~ line 425.

Response: The material referenced above has been moved to para 8 of the discussion.

Comment 16: The purpose of Experiment 3 unclear.

Response: The purpose of Experiment 3 was to demonstrate pathway specificity in the plasticity induced by optogenetic stimulation. This experiment was requested by a previous reviewer of an earlier version of this paper to test if the LTD and LTP protocols altered mPFC response specifically to the thalamic input without altering the response to other glutamatergic afferents, such as the ventral hippocampus. This has been clarified in the results section for experiment 3.
